# Oral lesions in Sjögren’s syndrome: A systematic review

**DOI:** 10.4317/medoral.22286

**Published:** 2018-06-21

**Authors:** Julia Serrano, Rosa-María López-Pintor, José González-Serrano, Mónica Fernández-Castro, Elisabeth Casañas, Gonzalo Hernández

**Affiliations:** 1 Department of Oral Medicine and Surgery, School of Dentistry, Complutense University, Madrid, Spain; 2 Rheumatology Service, Hospital Infanta Sofía, Madrid, Spain

## Abstract

**Background:**

Sjögren’s syndrome (SS) is an autoimmune disease related to two common symptoms: dry mouth and eyes. Although, xerostomia and hyposialia have been frequently reported in these patients, not many studies have evaluated other oral manifestations. The aim of this systematic review was to investigate prevalence rates of oral lesions (OL) in SS patients and to compare it to a control group (CG), when available.

**Material and Methods:**

An exhaustive search of the published literature of the Pubmed, Scopus, Web of Science and the Cochrane Library databases was conducted according to the Preferred Reporting Items for Systematic Reviews and Meta-Analyses Protocols (PRISMA-P) for relevant studies that met our eligibility criteria (up to September 1st 2017).

**Results:**

Seventeen cross-sectional studies and one cohort study were finally included. The results showed that SS patients presented more OL compared to non-SS patients. The most frequent types of OL registered in primary and secondary SS were angular cheilitis, atrophic glossitis, recurrent oral ulcerations and grooves or fissurations of the tongue, also when compared to a CG.

**Conclusions:**

OL are common and more frequent in SS patients when compared to a CG. This may be a consequence of low levels of saliva. More studies where these OL and all the possible cofounding factors are taken into account are needed.

** Key words:**Sjögren’s syndrome, oral lesions, oral diseases, oral manifestations, oral disorders, systematic review.

## Introduction

Sjögren’s syndrome (SS) is one of the most frequent autoimmune rheumatic diseases. It affects 0.5-1% of the population, occurring more middle-aged women than in men, with a ratio of 9:1 (1). Although it can appear at any age, it usually arises between the fourth and sixth decade of life. SS is a systemic exocrinopathy of unknown aetiology, which mainly affects the lacrimal and salivary glands giving rise to dry eyes and hyposalivation. It may manifest as primary SS (pSS), which occurs as an isolated disease, or as secondary SS (sSS) when it appears simultaneously with other autoimmune disease (1-3). There have been many classification criteria suggested for pSS (4,5). Nowadays, the most widely used is the one proposed by the American-European Consensus Group in 2002 (6). Other diagnosis criteria also accepted are the ones proposed by the American College of Rheumatology and the Sjögren’s International Collaborative Clinical Alliance for pSS (7).

Saliva has an important role in preserving oral health. Therefore, hyposalivation (or hyposialia) frequently increases the risk for different oral problems such as tooth decay, periodontal disease or fungal infections (8-13). Tongue alterations and non-specific ulceration have also been reported (9,14). The association between SS and oral lesions of autoimmune aetiology as lichen planus, recurrent aphtous stomatitis, pemphigus vulgaris and mucous membrane pemphigoid remains unclear (15).

This is the first systematic review that unifies all the oral lesions (OL) -non-xerostomia and/or hyposalivation- shown in the SS patients. The objective of the present study was to evaluate which OL are the most frequent in SS patients and compare them with a control group (CG). Knowing this, future dental protocols could be carried out, with the aim of improving SS patient’s oral health and quality of life.

## Material and Methods

This systematic review was conducted according to the Preferred Reporting Items for Systematic Reviews and Meta-Analyses Protocols (PRISMA-P) 2015 statement (16).

-Focused question

Based on the PRISMA guidelines, 2 focused PICO (population, intervention, comparison, and outcome) questions were constructed: 1) Which are the most frequent OL (non-xerostomia and/or hyposalivation) in SS patients? 2) Do SS patients have a higher prevalence of these OL when compared to a CG?

-Search Strategy

**Figure 1:**
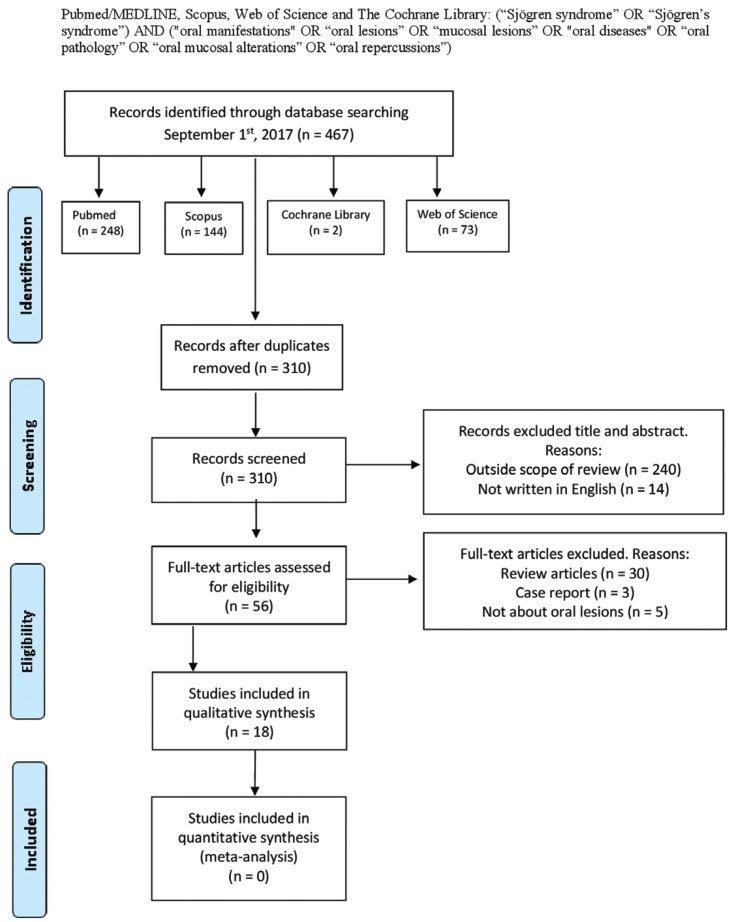
Flow diagram of the literature search, according to the Preferred Reporting Items for Systematic Reviews and Meta-Analyses (PRISMA).

A comprehensive search of the scientific literature was conducted without date restriction until September 1st 2017, in the following databases: PubMed/MEDLINE, Scopus, Web of Science and The Cochrane Library by two independent researchers (JS, JGS). The search strategy used was: (“Sjögren syndrome” OR “Sjögren’s syndrome”) AND (“oral manifestations” OR “oral lesions” OR “mucosal lesions” OR “oral diseases” OR “oral pathology” OR “oral mucosal alterations” OR “oral repercussions”) according to each database (Fig. 1). Furthermore, an additional hand search was performed to find potential eligible studies as reference lists of review articles and relevant studies.

-Study selection 

•Inclusion criteria. Full-text articles were included regardless of time period of study and year of publication.

Types of studies. The studies included had to be (a) original articles published in scientific journals, (b) cross-sectional or cohort studies, (c) comparative studies (SS group and CG), if available, (d) only in humans, and (e) written in English language.

Types of population. Individuals with SS that could have pSS and sSS (no restriction for SS diagnosis classification criteria was applied). CG population had to be healthy patients.

Outcomes. We considered oral alterations, oral manifestations and oral repercussions as OL. Neither xerostomia nor hyposialia were included as OL. In addition, we did not include dental lesions or periodontal disease. We considered oral candida lesions when clinical changes, such as angular cheilitis, atrophic glossitis, erythematous candidiasis, pseudomembranous candidiasis, or median rhomboid glossitis were described. We did not consider only positive cultures as OL. The studies must evaluate the presence of oral mucosal lesions and specify the number and/or percentage in the SS group, and the CG, if available.

•Exclusion criteria. (a) Those articles published in a language other than English, and (b) review articles, experimental studies, case reports, commentaries and letters to the Editor.

-Data collection and extraction 

Two independent researchers (JS and JGS) compared search results to ensure completeness and then duplicates were removed. Both reviewers individually screened all full title and abstract of the identified articles. Differences in eligible studies were resolved by discussion with a third reviewer (RMLP). Relevant full-text articles were obtained, and checked for eligibility using the following standard abstraction forms: first authors, journal, country in which was conducted, title of the paper, type of study, recruitment of patients, sample characteristics (population, age, and gender of SS patients and CG, when available), type of SS, diagnosis criteria for SS, and oral mucosal diseases diagnosis criteria ( Table 1,  Table 1 continue). In  Table 2,  Table 2 continue, we reported the prevalence of the different OL in SS patients and CG and, the statistical signification if there was CG, and it was available.

**Table 1:**
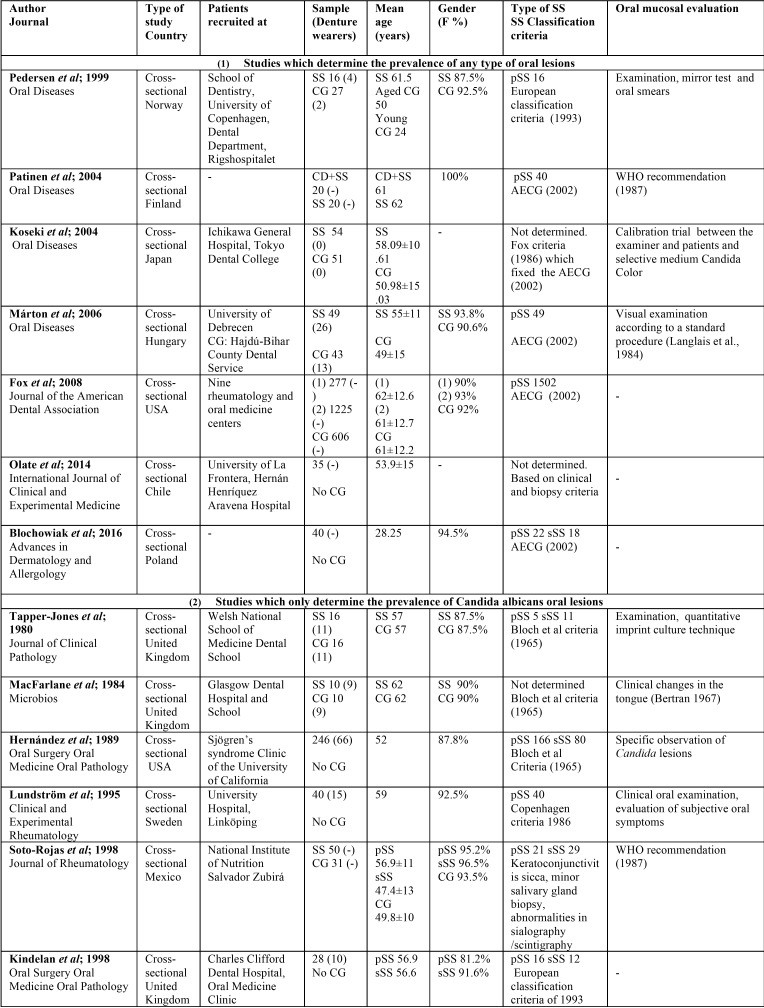
Study characteristics.

**Table 1 continue:**
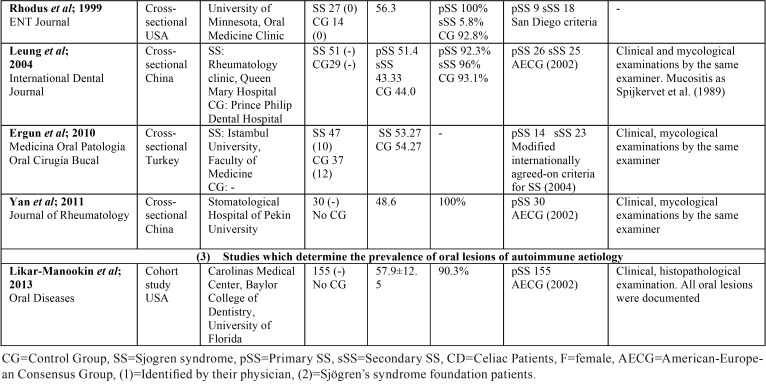
Study characteristics.

**Table 2:**
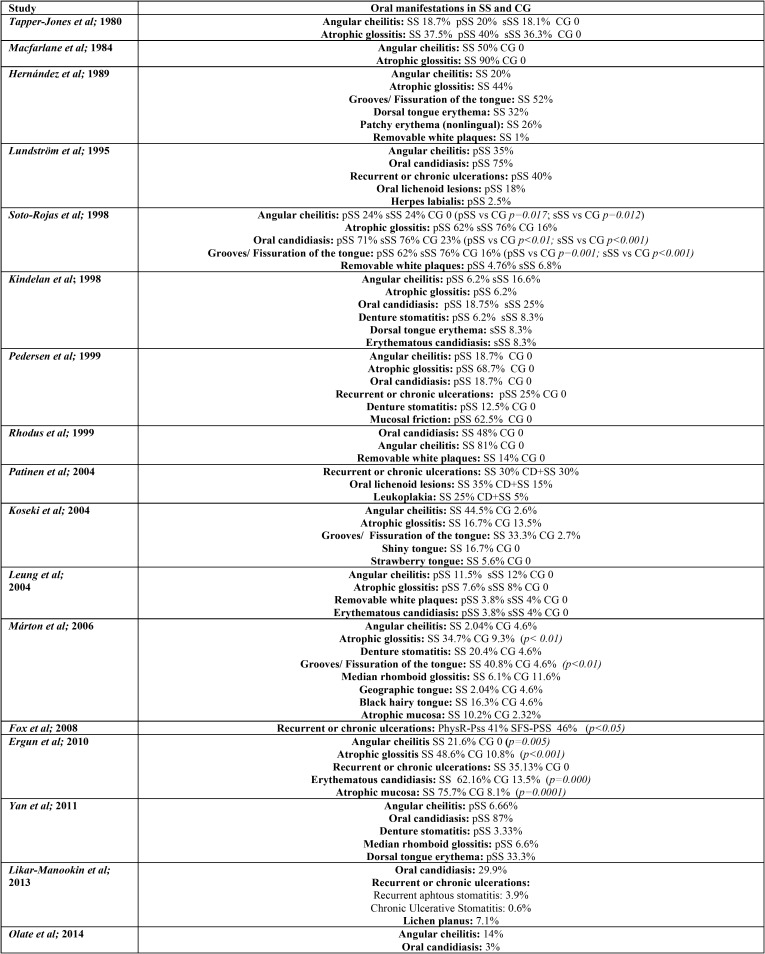
Oral manifestations in SS and CG.

**Table 2:**
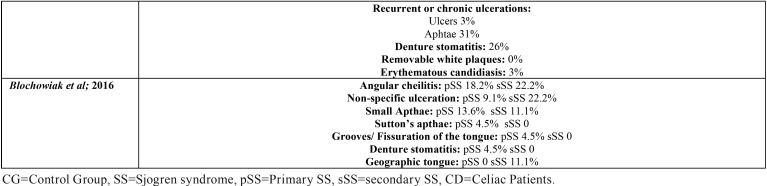
Oral manifestations in SS and CG.

-Quality Assessment

The Joanna Briggs Institute Prevalence Critical Appraisal Tool (JBI) for Studies Reporting Prevalence Data (17) was used to evaluate the methodological quality of the selected studies ( Table 3).

**Table 3:**
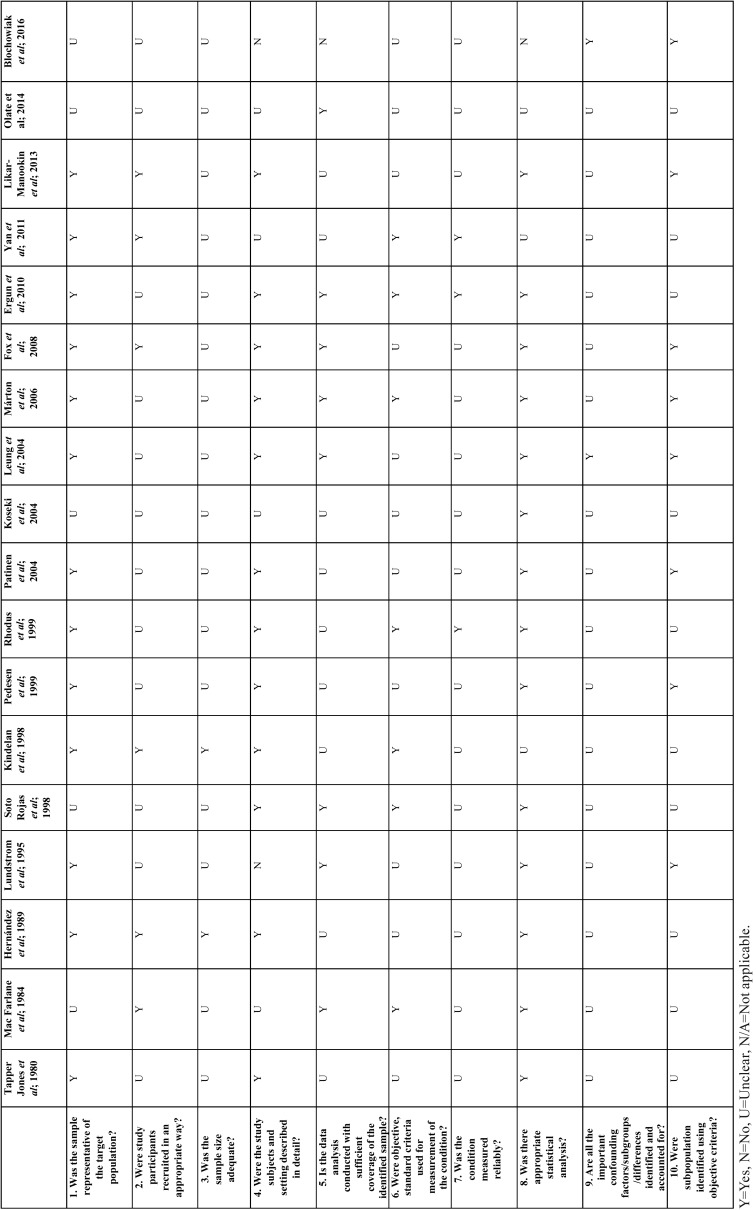
Risk of bias according to the JBI.

A study was considered to have a low quality assessment if a 0-5 criteria was met, and high quality assessment if studies met 5-10 criteria. Two reviewers (JS and JGS) conducted independently a critical appraisal, comparing and discussing afterwards their results. If the two reviewers disagreed on the final critical appraisal, a third reviewer (RMLP) was required.

-Categorization of Studies

In order to clarify the results, we categorized the studies in different groups: 1) studies which determine the prevalence of any type of OL, 2) studies which only determine the prevalence of Candida albicans lesions and 3) studies which determine the prevalence of OL of autoimmune aetiology.

-Data items and synthesis of results

The prevalence of oral mucosal lesions from the included studies was presented as a percentage. This percentages and their statistical significance, when available, shown along with the number of SS and CG (when available), were recorded in  Table 2,  Table 2 continue. A meta-analysis was not possible to carry out due to the differences between the selected papers: different types of SS, different SS diagnosis criteria, lack of agreement in OL diagnosis, and absence of healthy CG in some of the studies.

## Results

-Study selection

The search strategy yielded 467 results, of which 310 remained after removing duplicates. We screened all the titles, excluding those written in any language other than English, and those that were out of scope of review, obtaining a total of 56 eligible publications. Then, two independent researchers (JS and JGS) reviewed all the titles and abstracts, and excluded those that were reviews, case reports or did not specify oral disorders. Due to the study populations in the papers carried out by Soto-Rojas (14,18) were exactly the same (with the same result data) we considered both publications as only one article in order to unify the oral manifestations. The same resolution was taken for those carried out by Rhodus (19,20). Thirty-six studies, which did not fulfil the eligibility criteria, were excluded (Appendix 1). Finally, 18 articles were included in our systematic review (3,9,11-15,19,21-30) (Fig. 1).

-Study characteristics

Seventeen of the eighteen selected articles were cross-sectional studies and the other one was a cohort study. They were published between 1980 and 2016. A total of 3290 patients were studied: 2426 were SS patients (of which known: 2111 had pSS and 216 sSS), 3 of the studies did not specify the type of SS (MacFarlane *et al.*, 10 SS patients; Koseki *et al.*, 54 SS patients; and Olate *et al.*, 35 SS patients), and 864 patients were CG ( Table 1).

The mean age of the subjects ranged from 28.25-62 years in the SS group and 24-62 years in the CG ( Table 1).

Regarding to gender, in the SS patients the female percentage ranged from 81.2% to 100%, and in the CG from 87.5% to 100%. Three articles did not specify the gender of the sample (12,28,30).

We did not consider the CG in Patinen *et al.* study, since they were celiac patients; neither in Kindelan *et al.* study (since they were xerostomic controls), nor Yan *et al.* (because they had oral candidiasis) ( Table 1).

-Main findings 

The most frequent OL among SS patients was angular cheilitis, reported in fifteen of the eighteen selected papers. Atrophic glossitis was also common, reported in ten of the selected papers. Candida manifestations and recurrent or chronic oral ulcerations in eight of them; and grooves or fissuration of the tongue were reported in seven papers. None of the selected papers reflected the total prevalence within the SS or the CG patients ( Table 2).

This is in accordance with what we found when compared to a CG. The types of OL which were significantly more common in SS are: angular cheilitis, (14,28) atrophic glossitis (9,28), grooves or fissuration of the tongue (9,14), clinical manifestation of candidiasis (14), erythematous candidiasis (28) and atrophic mucosa (28). Oral manifestations, with its respective percentages, both in SS and CG patients are recorded in  Table 2.

-Risk of bias in individual studies

Using the predetermined 10 domains for the methodological quality assessment according to the JBI (17), we determined ten of the selected papers (3,11,12,14,21,22,25,26,29,30) to have a low quality assessment and eight of them (9,13,15,19,23,24,27,28) to have a high quality assessment. Table 3 shows a more detailed description of the articles included.

-Risk of bias within studies 

We detected some sources of information bias. First of all, different diagnosis criteria for SS have been used along the years. Second of all, some studies did not specify how the oral mucosal evaluation was carried out (3,13,19,24,30). Six studies (3,11,23,24,29,30)did not compare the outcomes with a healthy CG and three studies did not specify the gender of the sample nor the CG (12,28,30). In addition, three studies did not determine the type of SS studied (12,22,30). The studies did not take into account the presence of confounding factors as smoking and alcohol habits, other diseases or drugs intake, and eight of them did not report if the patients wore dentures (3,13-15,26,27,29,30).

-Risk of bias across studies

Due to the fact that only articles published in English were reviewed, bias due to language publication could not be ruled out. Even though we searched four databases, we cannot guarantee that some related papers might not have been identified. Additionally, not all OL were classified in the same way, and not all the studies specified if such lesions were reported by a calibrated (or always the same) examiner.

## Discussion

-Summary of evidence

SS is known to be one of the most common rheumatic diseases. To date, there is not a global overview of which are the most common OL in these patients, neither if they appear more frequently in SS than in healthy population.

-Main findings

We identified 18 studies reporting prevalence of oral mucosal lesions in SS, 10 of them compared to a healthy CG. We found surprising the young age of the patients. This is due to Pedersen *et al.* study consider a young CG, with a mean age of 24, and Blochowiak *et al.*, a study group with a mean age of 28.5. The rest of the papers, have a mean age around 50-60 years, which is more in accordance with the mean age of this disease ( Table 1).

In this systematic review, OL were more common among SS patients compared to non-SS patients. Angular cheilitis was the most frequent OL in SS patients, followed by atrophic glossitis; candida lesions; ulcers and grooves or fissuration of the tongue ( Table 2,  Table 2 continue).

When compared to a CG, the types of OL that appeared more frequently in SS with a statistical signification were also angular cheilitis; (14,28) clinical manifestation of candidiasis; (14) erythematous candidiasis;(28) atrophic mucosa; (28) atrophic glossitis (9,28) and grooves or fissuration of the tongue (9,14). These two last tongue alterations are characteristic signs of oral mucosal desiccation (9).

Geographic tongue was reported in two of the included papers (3,9). Less frequent tongue alterations were shiny tongue, strawberry tongue (12), and black hairy tongue (9) ( Table 2,  Table 2 continue). These tongue conditions, despite the discomfort that they cause, uncommonly require treatment.

The association between SS and OL of autoimmune aetiology remains unclear. Likar-Manookin *et al.* conducted the first study of autoimmune oral diseases in pSS. This study observed that 12.3% of pSS patients presented autoimmune OL such as lichen planus (7.1%) and recurrent aphtous stomatitis (3.9%). Chronic or recurrent ulceration seem to be common among SS patients: Lundström and Lindström reported a prevalence of 40%, which is in accordance with Fox *et al.* (43%); Ergun *et al.* (35.1% of oral ulcerations in the SS group vs 0% in the CG); Pedersen *et al.* (25%); and Patinen *et al.* (30%). Olate *et al.* differentiate between ulcers (3%) and aphtae, with a higher prevalence: 31%; and Blochowiak *et al.* classify them in non-specific ulceration (9.1% pSS, 22.2% sSS), small aphtae (13.6% pSS, 11.1% sSS), and Sutton’s aphtae (4.5% pSS, 0% sSS). In these papers the possible aetiology of these ulcerations was not given ( Table 2,  Table 2 continue).

Less frequently reported were oral lichenoid lesions (18-35%) (11,26), herpes labialis (2.5%) (11) and oral mucosal friction (62%) (25).

-Secondary data

The increased prevalence of OL in SS may be due to the impaired salivary gland function in these patients (25). Proper levels of saliva allow for lubrication of the mucosa, enhance healing of damage tissues, and play an essential role in local immunity (10,15,19). Additionally, Pedersen *et al.* found that oral mucosal changes occurred more frequently in patients with the lowest salivary flow rates.

It seems to be an inverse relationship between the rate of salivary flow and the presence of candidiasis: low levels of saliva are related to the presence of candidiasis (12,15,29,30). Kindelan *et al.* and Yan *et al.* found a significant inverse relationship between unstimulated salivary flow and Candida infection. Pseudomembranous candidiasis or removable white plaques was reported by five authors (18,19,23,27,30). We found interesting the fact that among SS patients pseudomembranous candidiasis was not common, with a prevalence range in the cited articles of 0%-6.8%. Denture wearing is one of the major predisposing factors for oral candidiasis, since the fitting surface of the denture is the main reservoir of the yeast (28). Nevertheless, neither Soto-Rojas *et al.* nor Pedersen *et al.* found a direct relationship between the presence of oral candidiasis and the use of dentures in SS patients.

-Strength and limitations

In order to carry out this systematic review, we conducted a specific search strategy for study selection. We included only those studies reporting prevalence of OL within the SS patients and, when available, those that compared them with a healthy CG. The comparison of the studies was limited due to the high degree of heterogeneity of OL. Although four databases were searched, we cannot rule out having missed relevant studies, also due to publication bias.

Diagnosis criteria of SS have changed periodically among the years. Since we did not have publication time restriction, different diagnostic criteria has been analysed among the reviewed studies. This must be taken into consideration when interpreting the results.

## Conclusions

In summary, the results of this systematic review showed that the prevalence of oral mucosal lesions in SS patients is higher than in non-SS patients. Angular cheilitis, oral manifestations of candidiasis, ulcerations, atrophic glossitis and grooves or fissuration of the tongue were the most reported lesions. When compared to a CG, the same lesions mention before appeared more frequently in SS patients. Some of these lesions (angular cheilitis, oral manifestation of candidiasis, groves or fissuration of the tongue) seem to be related to the impaired salivary gland function: low levels of saliva predispose to these kind of OL. Nevertheless, the relationship of other autoimmune OL as ulcerations remains unclear. This type of lesions may be directly attributed to SS and not necessary secondary results of the hyposialia. The clinician should know which the most frequent OL in SS patients are, in order to carry out dental protocols with the objective of preventing, diagnosing and treating them correctly, and therefore, improve the quality of life of SS patients.

Owing to the high degree of heterogeneity regarding the types of SS, diagnosis criteria of SS, and different diagnosis criteria of OL, it was difficult to compare the studies. In addition, the quality assessment showed the low quality of most of the existing studies. In our opinion, it is necessary to collect other risk factors in these types of studies such as alcohol or smoking habits, presence of removable prosthesis, oral status, systemic diseases, and drugs intake; considering that these factors could be also related to the presence of oral diseases. The majority of the studies reviewed, only determined the presence of Candida albicans oral manifestation. Therefore, we recommend that new studies in which a complete oral mucosal evaluation, looking for all possible OL ought to be carried out.
